# GOING BOLDER: PROMOTING DEMENTIA INFRASTRUCTURE THROUGH INTERACTIVE AND AI WEB-BASED TOOLS

**DOI:** 10.1093/geroni/igae098.1780

**Published:** 2024-12-31

**Authors:** Nicole Ruggiano, Monica Herzog, Zhe Jiang, Ellen Brown, Vagelis Hristidis, Jane Daquin

**Affiliations:** University of Alabama, Tuscaloosa, Alabama, United States; University of Alabama, Tuscaloosa, Alabama, United States; University of Florida, Gainsville, Florida, United States; Florida International University, Miami, Florida, United States; University of California Riverside, Riverside, California, United States; University of Alabama, Tuscaloosa, Alabama, United States

## Abstract

In 2018, the U.S. Congress successfully passed the Building Our Largest Dementia (BOLD) Infrastructure for Alzheimer’s Act to advance public health initiatives that support people living with dementia (PLWD) and their families. Part of the goals of BOLD include advancing community awareness about the latest research on dementia and support the translation of research findings to support PLWD, their families, and providers in addressing needs related to dementia care and caregiving. Infrastructure that supports information seeking is vital, given that caregivers often report challenges in finding the information they need about dementia, caregiving, and existing support. This is especially true for those who turn to the internet for information, where web-based search engines generate an overwhelming number of results to common questions posed by caregivers. To address these information needs, our interdisciplinary team has launched the Alabama Caregiver Connect initiative, which aims to more effectively link caregivers with information they need about dementia and caregiving by harnessing web-based tools that are interactive and driven by artificial intelligence. This presentation will review innovative technologies that the team has developed, implemented, and/or evaluated for their potential to support public health infrastructure for dementia. Findings presented will examine benefits and drawbacks of several approaches identified through the initiative, including ChatGPT, evidence-based chatbots, and crowdsourced asset mapping tools. Implications for policy and practice will be discussed.

